# Analysis of the Level of Stress and Methods of Coping with Stress among the Nursing Staff

**DOI:** 10.3390/nursrep13030111

**Published:** 2023-09-14

**Authors:** Anna Antczak-Komoterska, Beata Haor, Mariola Malinowska, Lech Grzelak, Monika Biercewicz, Dorota Kochman, Karolina Krajewska, Karolina Filipska-Blejder, Adam Wiśniewski, Robert Ślusarz

**Affiliations:** 1 Faculty of Health Sciences, State University of Applied Sciences in Wloclawek, 87-800 Wloclawek, Poland; 11108@puz.wloclawek.pl (M.M.); lech.grzelak@pans.wloclawek.pl (L.G.); d_kochman@poczta.onet.pl (D.K.); karolina.krajewska@pans.wloclawek.pl (K.K.); 2 Neurological and Neurosurgical Nursing Department, Faculty of Health Science, Collegium Medicum in Bydgoszcz, Nicolaus Copernicus University in Toruń, 85-821 Bydgoszcz, Poland; beata.haor@interia.pl (B.H.); karolina.filipska@cm.umk.pl (K.F.-B.); robert_slu_cmumk@wp.pl (R.Ś.); 3 Department of Geriatrics, Faculty of Health Sciences, Collegium Medicum, Nicolaus Copernicus University, 85-094 Bydgoszcz, Poland; kamamb@cm.umk.pl; 4 Department of Neurology, Faculty of Medicine, Collegium Medicum in Bydgoszcz, Nicolaus Copernicus University in Toruń, 85-094 Bydgoszcz, Poland; adam.lek@wp.pl

**Keywords:** stress, coping, nursing staff

## Abstract

In general, “stress” is the reaction of the body to mental and physical demands placed on it. Stress disrupts mental balance, and reduces the ability to work and function, which negatively affects the performance of duties. The aim of this study was to analyse the level of stress and ways of coping with it among nursing staff. The study covered 220 nurses employed at the Provincial Specialist Hospital in Włocławek. The research tool was the Perceived Stress Scale (PSS-10) and the Brief-COPE. The results of this survey showed the occurrence of average and high levels of experiencing stress in 36% and 40%of staff, respectively. Brief-COPE scale results show that substance use/gender (men) and use of emotional support/place of work (internal medicine department) are significant at *p* < 0.01. Considering the impact of the workplace on the use of psychoactive substances, it can be seenthat people working in the surgical ward are more likely to use psychoactive substances. Furthermore, nurses in the surgical ward find it easier to think and plan what to do when faced with a difficult life situation. Most often, the respondents with the highest work experience, i.e., the elderly, declared a return to religion. The results of the research indicate that the nursing community experiences stress to an average or significant degree. The strategies are mainly based on active coping and seeking emotional and instrumental support. Further research is needed in this field. This study was not pre-registered on a publicly accessibly registry.

## 1. Introduction

Each person experiences smaller or larger emotional tensions every day, which t cause stress. The way this affects people at work has generated a lot of interest. Much information indicates that work-related stress contributes to many heart diseases, stomach ulcers, migraines and mental disorders. This has major consequences for employees’ organizational behaviour and their family and personal lives. These diseases have significant economic and social costs [1,2,3]. The word “stress” is used almost every day. Most often, we use it to describe our malaise caused by a life situation. When we talk about stress, we mean stimuli that have caused changes in our lives and caused a stress reaction in us, although they are not always bad in themselves. Stress should not be understood as an unambiguously negative situation that needs to be fought. It stimulates action, to a moderate degree, allowing you to focus attention and make the right decision. It is only when it lasts too long that it contributes to a decrease in efficiency [4,5,6,7].

Stress is the body’s response to excess accumulated energy. When there is no way to find an outlet for the power at its disposal, it starts to become nervous. Generally speaking, the body is adapted to cope with stress, but only in small doses. Its excess is very harmful to both the body and the spirit [8].

Hans Hugo Selye (the doctor who introduced the concept of stress) asserts that stress is the body’s reaction in the form of mobilizing energy to overcome various obstacles, barriers, requirements, regardless of whether it is accompanied by pleasant or unpleasant feelings. It is a non-specific reaction, i.e., its type does not depend on the type of factors that causes it [9,10].

The most important thing is to distinguish whether stress is positive or beneficial (eustress) or negative or harmful (distress). When our body mobilizes to take action and when we act effectively and rationally thanks to this, motivation is high—we are talking about stress. However, when stress is too much, emotions are too high; after crossing the optimal point, stress begins to harm us, makes our actions and behaviour disintegrate, and consequently leads to exhaustion.

In the theories of stress, biological stress and psychological stress are distinguished. In healthy people, a natural reaction due to the occurrence of psychological stress is to strive to reduce tension, i.e., to try to cope. According to Lazarus and Folkman, coping means “constantly changing cognitive and behavioural efforts aimed at mastering specific external and internal requirements, assessed by a person as taxing or exceeding their resources” [11,12].

A person adopting a problem-oriented style undertakes various types of action aimed at solving a given problem or changing a stressful situation in the form of cognitive processes. These are people who perceive the problem task-wise. They assess the situation without emotional involvement, search for information in order to solve it in the best possible way, and have the ability to adapt to acting in changed conditions. People who adopt an emotion-focused style tend to focus on themselves and their own experiences. They undertake struggles that lead to the reduction in emotional tension, and very often they are unfavourable towards the existing problem, which results in greater stress [12,13,14,15,16,17,18].

The literature shows that both stress and stress-coping strategies are of great interest to researchers. Research by Mao et al. [19] and Tam et al. [20] show that high work intensity can ultimately lead to burnout. Mindfulness-based intervention has been concluded to be effective in promoting mental health and reducing the effects of burnout. Stress is an inseparable element of the nurse’s work, but the task of the management staff is to minimize the negative emotions experienced. The authors provide several solutions to improve the situation. Among other things, mindfulness workshops for healthcare professionals, including students on the ward, to reduce their stress and promote mental well-being.

The objective of this study was to investigate the level of stress and methods of coping with stress among the nursing staff in the department of internal medicine and the surgical department.

## 2. Materials and Methods

### 2.1. Study Design and Participants

From November 2020 to August 2021, we conducted this hospital-based cross-sectional study in the Provincial Specialist Hospital in Włocławek in the internalorsurgicaldepartments. This research was anonymous. The sample consisted of 220 male and female nurses, aged 22 to 60. Participants had to meet the following inclusion criteria:

− Six months of working experience in a hospital;
− Working full-time and providing direct care for patients from internal medicine and surgical departments;
− Persons who agreed to participate voluntarily were included in the study.

The research was carried out in the wards. At the beginning of the study, the subjects received basic information about the course of the study and the rules in force during its duration. The studied person had the right to refuse to participate in the study. After obtaining consent, the nurses received a questionnaire from one of the researchers. Then, after completing the survey, respondents placed it in a specially prepared, sealed urn, located in a designated place.

### 2.2. Measures

The PSS-10 questionnaire (Perceived Stress Scale) was used to assess the intensity of perceived stress [21]. The Polish version of the PSS-10, introduced by Juczyński and Ogińska-Bulik in 2009, was employed. Participants were asked to respond on a 5-point Likert scale (1  =  never, 2  =  rarely, 3  =  sometimes, 4  =  often, and 5  =  always) to a total of ten items reflecting the frequency of their stress symptoms. Internal consistency was tested in a study of 120 adults with a Cronbach’s alpha of 0.86 [22]. This tool consists of 10 questions about thoughts and feelings related to experiences in the last month. The higher the score, the greater the stress level. The cut-off point for the PSS-10 scale was 24 points, as the average level of perceived stress.

The second tool used in the study was the Coping Orientation to Problems Experienced Inventory (Brief-COPE) [23], which is designed to assess coping with stress and ways of reacting to difficult situations. Most often, this method is used to measure dispositional coping, i.e., the assessment of reacting to and experiencing strong stress. This tool is self-descriptive and is used for individual and group studies. It consists of 28 statements included in 14 strategies (2 statements in each strategy): self-distraction (items 1 and 19), active coping (items 2 and 7), denial (items 3 and 8), substance use (items 4 and 11), use of emotional support (items 5 and 15), use of instrumental support (items 10 and 23), behavioural disengagement (items 6 and 16), venting (items 9 and 21), positive reframing (items 12 and 17), planning (items 14 and 25), humour (items 18 and 28), acceptance (items 20 and 24), religion (items 22 and 27), and self-blame (items 13 and 26). The internal consistency of the Polish version of Brief-COPE was established on the basis of a survey of 200 people aged 25–60. Thes Split-half reliability coefficient for this scale was 0.89 (Guttman’s coefficient was 0.87) [20]. In Poland and worldwide, these scales have been recognized as reliable and valid tools, which proves their very good psychometric properties.

### 2.3. Ethical Statement

The study was approved by the Bioethics Committee of the State Vocational University in Włocławek, by the Director of the Provincial Specialist Hospital in Włocławek. The study was conducted according to the Declaration of Helsinki regarding research on humans. All subjects provided informed consent to participate in the study.

### 2.4. Statistical Analysis

The statistical analysis was performed with STATISTICA version 13.1 (Dell Technologies, Round Rock, TX, USA). The Mann–Whitney U test was used to assess differences in the level of stress and perceived stress in two populations (gender, place of residence and place of work), while in the case of more than two populations (age, education and work experience) the Kruskal–Wallis ANOVA test was used. The test PSS-10 result was converted to the sten scale. The sten scale is a psychological test scale normalized so that the population mean is 5.5 and the standard deviation is 2. There are 10 units in the scale. The result of 1–4 sten is considered low, 5–6 sten is considered average, and 7–10 sten is considered high. A *p*-level <0.05 was considered statistically significant.

## 3. Results

The study group consisted of 179 women, who constituted 81.4% of the respondents and 41 men, who constituted 18.6%. The respondents were grouped into four age groups: 22–30 (7.3%), 31–40 (68.6%), 41–50 (40.5%) and 51–60 (25.5%). The vast majority of the respondents, as many as 68.60% lived in the city; the rest were inhabitants of the countryside. Among the respondents, 94 people graduated with a bachelor’s degree in nursing, 60 people had a master’s degree in nursing and the rest were certified nurses. The respondents were also differentiated in terms of work experience: 7.7% had worked in the profession for 1–5 years, 11.8% for6–10 years, 31.8% of a given group indicated 11–20 years of work experience and 20.5% indicated the longest, i.e., 31–40 years of work. The last criterion for the division of the study group was the indication of the ward for their current work: over 51.8% performed their profession in the internal medicine department and 48.2% in the surgical ward. Detailed sociodemographic data are presented in Table 1.

In life, there are often stressful situations, so the respondents were asked how they feel and how they cope in nervous situations. When asked how often in the last month they were nervous because something unexpected happened, only 1 person did not feel nervous, 92 people sometimes felt nervous, and as many as 83 people felt stress quite often. More than half of the surveyed group during the last month felt that sometimes important things in their lives got out of hand. On the other hand, 105 people, constituting less than 50% of the study group, sometimes felt nervous and tense at work. More than 30% of the nurses surveyed during the last month were sometimes or quite often convinced that they were able to cope with personal problems. Fewer than 42% of the respondents sometimes felt that things were going their way and 32% felt this way quite often. During the survey, 58.18% of the surveyed population stated that during the last month they sometimes did not cope with all their duties, and fewer than 2% had no problems in performing the tasks. Fewer than half of the surveyed health care workers, constituting approx. 49%, were able to control their irritability very often, and approx. 45% felt that sometimes everything works out for them. During the study period, 136 people declared that they sometimes became angry because they had no influence on what happened and 135 people sometimes felt that they could not overcome the increasing difficulties. The level of perceived stress determined using the PSS-10 scale is presented in Table 2.

The average general PSS-10 index reached the value of 24.5 ±3.5., with 35.9% of respondents achieving an average result, 24.5% of them achieving a lower result, i.e., they are less exposed to stress than others, and 39.57%scoring higher, i.e., they are more exposed to stress than others. The minimum value obtained is 12. The maximum value obtained is 40.

The results of the Mann–Whitney U test indicate that the gender, place of residence and place of work did not differentiate the general level of stress perceived by medical personnel. The mean values in the scope of the examined variables were similar in the analysed groups. Also, the results of the Kruskal–Wallis test did not show statistically significant differences between age, education and work experience in terms of the general level of stress (Table 3).

According to theBrief-COPE questionnaire, we find that 41.8% (92 people) of the surveyed population, when in a very difficult situation, often engage in work or other activities in order not to think about the problem. However, the next question shows that about 42% of the respondents always focus their efforts on solving a difficult situation and seriously wonder what steps should be taken. The respondents (48.6%) rarely lie about the reality, telling themselves that a difficult situation is untrue, and about 80% almost never drink alcohol or take other drugs to feel better and to manage the problem more easily. Almost half of healthcare professionals (approx. 45%) receive emotional support, help or advice from others during difficulties. When a difficult situation arises, about 43% of respondents almost always take action to improve the situation, and about 41% always try to develop a strategy or plan specifying what should be done. More than 50% of the surveyed group rarely refuse to believe that they are really in a difficult situation and about 46% rarely talk about things that allow them to escape unpleasant feelings. The analysis of the survey shows that about 40% almost never or rarely give up trying to achieve the goal and cope with it. A total of 93 people answering questions when they have problems almost always seek advice and help from others on what to do, and 105 people declared that they receive reassurance and understanding from others. Among the respondents, about 47% rarely joke about problems and about 72% rarely treat them as fun. Often the surveyed respondents (44%) do something to think less about a difficult situation, e.g., go to the cinema, watch TV, read, daydream, sleep or go shopping. More than half of the respondents often blame themselves for what happened and criticize themselves, and often try to find solace in religion or another faith by praying or meditation. A total of 50.5% of respondents often try to see the problem in a different, more positive light, and about 41% often look for the good in what happened. A rarely surveyed group (44.1%) accept the fact that it has already happened and almost never or rarely reveal its negative emotions. Finally, more than 40% rarely and fewer than 40% often learn to live with this difficult situation (Table 4).

The strategies of coping with stress most often used by the respondents are primarily seeking emotional support, an average of 2.23 points, then active coping and seeking instrumental support—an average of 2.22 points, and planning—an average of 2.20 points. Respondents used such strategies as the following the least: turning to religion and blaming oneself—average 1.07 points, cessation of activities—average 0.89 points, sense of humour—average 0.56 points and using psychoactive substances—average 0.28 points. Another tool used was the EEP-10’s questionnaire, with which the intensity of perceived stress was assessed (Table 5.).

In life, there are often stressful situations, so the respondents were asked how they feel and how they deal with nervous situations. When asked how often during the last month you were upset because something happened unexpectedly, only 1 person did not feel nervous, 92 people were upset sometimes, but enough, and as many as 83 people often felt stress. More than half of the study group during the last month felt that sometimes the important things in their lives were out of their control. On the other hand, 105 people, constituting less than 50% of the study group, sometimes felt nervousness and tension at work. Over 30% of surveyed nurses and 41 nurses in the last month sometimes or quite often were convinced that they were able to deal with personal problems. Fewer than 42% of respondents sometimes felt that things were going their way and 32% felt that way quite often. During the survey, 58.18% of the surveyed population stated that during the last month sometimes they could not cope with all the duties and fewer than 2% had no problem in performing tasks. Fewer than half of the surveyed health service employees, constituting about 49%, were able to control their irritability very often, and about 45% felt that sometimes everything works out for them. During the period under review, 136 people declared that they sometimes became angry because they had no control over what happened and 135 people sometimes felt that they could not overcome the increasing difficulties.

Another element that has been checked is the impact of sociodemographic factors on strategies for coping with stress, which was analysed on the basis of the following Table 6 and Table 7. Table 6 shows that substance use/gender and use of emotional support/place of work are significant at *p* < 0.01. The survey shows that men declared a tendency to use psychoactive substances much more often among the surveyed respondents. For respondents working in the internal medicine department, the use of emotional support, and understanding and encouragement during life’s difficulties, were encountered more than with nurses working in the surgical ward. In turn, substance use/place of work, planning/place of work and religion/work experience are significant at *p* < 0.05. Considering the impact of the workplace on the use of psychoactive substances, it can be seen that people working in the surgical ward are more likely to use psychoactive substances. Furthermore, nurses in the surgical ward find it easier to think and plan what to do when faced with a difficult life situation. Most often, the respondents with the highest work experience, i.e., the elderly, declared a return to religion.

## 4. Discussion

In Poland, nursing is one of the most numerous professional groups. The vast majority of European Union countries experience a large deficit among professionally active nursing staff [21]. Stress in the work of a nurse has long been identified with issues such as physical work, shift work, human suffering, contact with other staff and interpersonal relationships. Work is considered one of the most important forms of human activity. Professional work can contribute to the acquisition of various new skills and coping with new challenges. It brings with it a lot of positive emotions and satisfaction, although it is not always a source of satisfaction; quite often it is the cause of dissatisfaction or frustration [22,23,24]. In the presented work, an attempt was made to analyse the level of stress and ways of coping with it among the nursing staff. In life, stress is aninseparable element of every day; therefore, when examining the impact of sociodemographic factors on the assessment of the intensity of stress, the respondents were asked how they feel and how they cope in nervous situations [25,26,27,28,29]. When asked how often in the last month they were nervous, only 1 person did not feel nervous, 92 people sometimes felt nervous, and as many as 83 people felt stress quite often. In this study, the average PSS-10 scale index was 24.5 ± 3.5, with 35.9% of the respondents achieving an average result, 24.5% of them having a lower result, i.e., they are less exposed to stress than others, and 39.57% achieving a higher result, i.e., they are more exposed to stress than others.

According to the study conducted by Kowalczyk et al., the PSS-10 questionnaire showed that 55.4% of the surveyed male and female nursesfeel high levels of stress, and 31.2% feel average levels. Only 13.4% of respondents were not stressed by their profession [25]. According to the Śniegocki [26], the level of stress experienced by 44 nurses working in surgical departments was very similar to the results of Kowalczyk et al. [25]. A total of 16% of the respondents presented a low level of stress, 38% anaverage, and as many as 45% of the respondents presented a high level of stress. Similar results were also presented by Teixeira et al. [30] among Brazilian nurses, where as many as 75.5% declared that they experienced strong occupational stress.

The test used in the work was the Brief-COPE questionnaire, which includes 28 statements that are part of 14 coping strategies (2 statements in each strategy), which are: active coping, planning, positive re-evaluation, acceptance, sense of humour, turning to religion, seeking emotional support, seeking instrumental support, doing something else, denial, venting, using psychoactive substances, stopping activities, and self-blame. The most frequently used strategies for coping with stress by the study groups were seeking emotional support—average 2.23 points, then active coping and seeking instrumental support—average 2.22 points, and planning—average 2.20 points. The respondents used such strategies as the following the least: turning to religion and self-blame—average 1.07 points, cessation of activities—average 0.89 points, sense of humour—average 0.56 points and using psychoactive substances—average 0.28 points. Analysing the impact of sociodemographic factors on strategies for coping with stress, it should be said that it is statistically significant to state that gender and workplace influence the use of psychoactive substances [31]. However, the workplace itself affects the search for emotional support and planning. The influence of gender on the use of psychoactive substances is presented as follows: women—0.22, men—0.5. Among the respondents, men definitely more often declared a tendency to use psychoactive substances to alleviate unpleasant emotions during difficult life situations. Considering the impact of the workplace on the use of psychoactive substances, it can be seen that the average of 0.35 points was obtained by people working in the surgical ward, and people working in the preventive department received 0.2 points. In addition, respondents working in the preventive department seek and need emotional support, understanding, and comfort during the difficulties encountered in life more than nurses working in the surgical ward. This means that people working in the surgical ward find it easier to think and plan what to do when they encounter a difficult life decision. 

Research by Kowalczuk et al. [3] indicate that the choice of coping strategy is also influenced by demographic factors, such as age, education, having children, and marital status.

In the study by Kowalczyk et al. [25], the following methods of coping with stress prevailed among the nursing staff: active coping, planning, and seeking emotional and instrumental support. On the other hand, the methods least frequently used by the respondents turned out to be the following: turning to religion, sense of humour, cessation of activities, and taking psychoactive substances. In the study by Śniegockis [26], it was found that the most frequently used strategies among the respondents was the use of friends’ help (63%), which is classified as a form of emotional support, and the respondents declared drinking alcohol (9%) and taking psychoactive substances (2%) least often. Similar results were obtained by Jachimowicz-Wołoszynek et al. [32], whose treatment nurses strived for perfection, adopted offensive problem-solving strategies, and were characterized byahigh sense of social support. Also, studies by Teixeira et al. [30] showed that the most frequently used strategies among nurses of a Brazilian hospital were problem-focused strategies (60%) in the form of active coping. In the study by Stefanowicz-Bielska et.al., most nurses responded that they have often or almost always been concentrating their efforts on doing something about the situation they are in (45% and 42%, respectively) and they have been taking action to try to make the situation better (37% and 51%, respectively). The majority of nurses stated that they never take the following action: I’ve been saying to myself “this isn’t real” (48%), I’ve been using alcohol or other substances to make myself feel better (72%), I’ve been using alcohol or other drugs to help me get through it (75%), and I’ve given up the attempt to cope (51%) [33]. Analysing the impact of education in the presented work, we notice that staff with a diploma (1.69) and a master’s degree (1.7), more often than people with a bachelor’s degree (1.46), during difficult life situations deal with other activities in order not to think about the problem. In the studies by Kowalczyk I. [25] et al., nurses with vocational or secondary education more often coped with stress through acceptance and cessation of activities (*p* < 0.05). On the other hand, the results presented by Żuralska et al. [34] show that education has no significant impact on the style of coping with stress. In the study of the Śniegocki [26], people with higher education statistically more often use the strategy of planning and active coping with a stress factor. The factor analysis in this study in relation to seniority showed that the respondents with the highest seniority, i.e., older people (aged 31–40—1.21), most often declared a turn towards religion. A slightly lower indicator was indicated by people with 21–30 years of service (1.18) and 1–5 years (1.15). The least support in prayer or meditation during the emerging problem was sought by healthcare professionals with 6–10 years of experience (0.87). In the study by Kowalczyk et al. [25], the analysis of seniority showed that the greater the number of years worked in the profession, the more often the strategy focusing on the problem—planning (*p* < 0.05)—and strategies focusing on emotions—turning to religion (*p* < 0.05)—were used. The study by the Śniegocka and Śniegocki [26] shows that nurses with fewer than 20 years of experience in the profession used styles of behaviour significantly more often, which significantly differentiates the obtained results.

Analysing our own results with those presented by the authors, it can be assumed that they are the same in many respects. In the case of the most frequently used strategies of coping with stress by the study groups, the results were confirmed by Kowalczyk et al. [25]. A similar consistency can be seen in the study by Śniegoski [26] in terms of the lowest degree of strategy use. However, the results differed slightly from the studies conducted by Jachimowicz-Wołoszynek et al. [32] and Teixeira et al. [30]. In the case of the analysis of the impact of socio-demographic factors, the data confirmed the reports from the literature [25,26,34]. Recently, there has been a tendency to use stress coping strategies. Given the high rate of perceived stress, dealing with it reduces the risk of consequences, both for mental and physical health.

The correlation of active coping with the level of perceived stress shows that the higher the level of perceived stress by the surveyedmale and female nurses, the higher the indicator of active coping with stress and the undertaking of actions aimed at improving the situation. The correlation of the search for emotional support with the level of stress felt tells us that the more the respondents felt stress, the more they sought emotional support, understanding, and reassurance from others. The more the respondents felt the level of stress, the more they sought instrumental support, i.e., help and advice from others, and the more they perceived the emerging problem in a positive light. In addition, as the level of stress felt increased, so did the level of coping by thinking and planning what to do to solve the problem [35,36,37].

The results by Iwanowicz-Palus and the team revealed differences in sociodemographic variables in the identified types that characterise nurses. Education and length of service of the respondents were associated with their use of specific styles of coping with stress. Nurses belonging to the harmonious type had a higher level of education. The higher the educational level of nurses, the more often they used the task-oriented stress coping style [38].

Śniegocka M. et al., in their research, presented a correlation between the strategy of coping with stress and the age and education of the respondents. Nurses with higher education more often used strategies of active coping with stress and the strategy of planning. The research presented by Śniegocka presents the influence of age on the frequency of using the strategies of a sense of humour and avoidance of competitive actions. Moreover, it was shown that after the age of 40, the coping strategy of “turning to religion” increases, which results from the need for emotional support [26].

The study by the Kupcewicz of the assessment of stress intensity on the PSS-10 scale aimed at identifying the subjective feelings of the study subjects associated with personal issues and events and the methods of coping with them. It was shown that the mean perceived stress level was 17.4 (SD = 5.3) on a scale of 0 to 40 points over the past month [39].

The study has several limitations. First, the study design was cross-sectional. Thus, it is not possible to determine the causality of factors affecting the level of stress. It is therefore necessary to continue the longitudinal study in order to be able to create an accurate characterization of stress experienced by nurses and ways of dealing with it over time. Secondly, the sample size in this study can be considered relatively small. Finally, our research was conducted only in selected departments. It is necessary to expand the environment of the study group.

## 5. Conclusions

The work of a nurse is difficult; it is very complex and involves multi-tasking and dealing with illness and death every day, mixed with helplessness and hope, and leading to mental strain, which can lead to the abuse of alcohol or sedatives. Representatives of this profession often show a tendency to accumulate negative emotions. Growing stress causes various specific and non-specific changes in the human body, which occur in both physiological and psychological processes.

Preventing and dealing with the negative effects of stress is a prerequisite for maintaining high efficiency and job satisfaction. Nursing staff use various mechanisms that allow them to survive stress, minimize it or avoid it or change the situation to a less-stressful one. Long-term stress is negative, and each person has a certain ability to bear stress and cope with it. Resistance to stress factors plays a big role here—it is individual, and social situation and ties with other people are important for coping with stress.

Long-term stress, even if it is not at the highest level, as in the case of this study, can be a threat in the mental and physical spheres. Based on this and the results of the research, it can be concluded that this type of research allows for the monitoring of the level of stress and raising awareness of methods of dealing with it. Despite the popularity of this type of research, the undoubted advantage of this type of research is the regularity of conducting research on stress, its effects, and methods of dealing with it.

The results of the research indicate that the nursing community experiences stress in an average or significant degree. The strategies are mainly based on active coping, and seeking emotional and instrumental support. The conducted analysis confirms to a large extent the results of other researchers.

## Figures and Tables

**Table 1:** Descriptive characteristics.

Feature		Number	Percent
**Gender**	Female	179	81.40%
	Male	41	18.60%
**Place of residence**	Village	69	31.40%
	City	151	68.60%
**Age**	22–30	16	7.30%
	31–40	59	26.80%
	41–50	89	40.50%
	51–60	56	25.50%
**Education**	Registered nurse	66	30.00%
	Bachelor of nursing	94	42.70%
	Master of nursing	60	27.30%
**Work experience**	1–5 years	17	7.70%
	6–10 years	26	11.80%
	11–20 years	62	28.20%
	21–30 years	70	31.80%
	31–40 years	45	20.50%
**Place of work**	Internal medicinedepartment	114	51.80%
	Surgical department	106	48.20%

**Table 2:** The level of stress perceived by the respondents.

Number of Points (PSS-10)	Sten	Level of Experienced Stress	Number	Percentage
**below 16**	1	Low	4	2%
**16–19**	2		8	4%
**20**	3		13	6%
**21–22**	4		29	13%
**23–24**	5	Average	54	25%
**25**	6		25	11%
**26–27**	7	High	48	22%
**28**	8		19	9%
**29–31**	9		16	7%
**over 31**	10		4	2%

**Table 3:** The impact of socio-demographic factors on the level of stress perceived by the respondents.

Variable	U	H	*p*
**Gender**	3505	-	0.653
**Place of residence**	4929	-	0.520
**Place of work**	5399	-	0.171
**Age**	-	0.84	0.839
**Education**	-	1.46	0.482
**Seniority**	-	2.51	0.642

U—Mann–Whitney U statistic value, H—Kruskal–Wallis test, *p*—significance level.

**Table 4:** Detailed characteristics of the results of the Brief-COPE.

Number of Item	Strategies of Brief-COPE	Nurses’ Responses					
		I Haven’t Been Doing This at All	A Little Bit	A Medium Amount	I’ve Been Doing This a Lot	M	SD
		N (%)	N (%)	N (%)	N (%)		
1. I’ve been turning to work or other activities to take my mind off things.	Self-distraction	29 (13.2)	61 (27.7)	92 (41.8)	38 (17.3)	1.6	0.68
19. I’ve been doing something to think about it less, such as going to movies, watching TV, reading, day-dreaming, sleeping, or shopping.		28 (12.7)	68 (30.9)	97 (44.1)	27 (12.3)		
2. I’ve been concentrating my efforts on doing something about the situation I’m in.	Active coping	2 (0.9)	42 (19.1)	85 (38.6)	91 (41.4)	2.22	0.62
7. I’ve been taking action to try to make the situation better.		7 (3.2)	31 (14.1)	87 (39.5)	95 (43.2)		
3. I’ve been saying to myself “this isn’t real”.	Denial	56 (25.5)	107 (48.6)	39 (17.7)	18 (8.2)	1.18	0.67
8. I’ve been refusing to believe that it has happened.		30 (13.6)	113 (51.4)	63 (28.6)	14 (6.4)		
4. I’ve been using alcohol or other drugs to make myself feel better.	Substance use	177 (80.5)	32 (14.5)	8 (3.6)	3 (1.4)	0.28	0.57
11. I’ve been using alcohol or other drugs to help me get through it.		175 (79.5)	30 (13.6)	11 (5.0)	4 (1.8)		
5. I’ve been getting emotional support from others.	Use of emotional support	8 (3.6)	30 (13.6)	99 (45.0)	83 (37.7)	2.23	0.65
15. I’ve been getting comfort and understanding from someone.		8 (3.6)	25 (11.4)	81 (36.8)	106(48.2)		
10. I’ve been getting help and advice from other people.	Use of instrumental support	5 (2.3)	41 (18.6)	81 (36.8)	93 (42.3)	2.22	0.69
23. I’ve been trying to get advice or help from other people about what to do.		8 (3.6)	36 (16.4)	69 (31.4)	107(48.6)		
6. I’ve been giving up trying to deal with it.	Behavioural disengagement	82 (37.3)	83 (37.7)	49 (22.3)	6 (2.7)	0.89	0.7
16. I’ve been giving up the attempt to cope.		86 (39.1)	85 (38.6)	42 (19.1)	7 (3.2)		
9. I’ve been saying things to let my unpleasant feelings escape.	Venting	19 (8.6)	102 (46.4)	81 (36.8)	18 (8.2)	1.1	0.57
21. I’ve been expressing my negative feelings.		96 (43.6)	85 (38.6)	35 (15.9)	4 (1.8)		
12. I’ve been trying to see it in a different light, to make it seem more positive.	Positive reframing	7 (3.2)	69 (31.4)	111(50.5)	33 (15.0)	1.76	0.63
17. I’ve been looking for something good in what is happening.		6 (2.7)	85 (38.6)	42 (19.1)	7 (3.2)		
14. I’ve been trying to come up with a strategy about what to do.	Planning	6 (2.7)	39 (17.7)	85 (38.6)	90 (40.9)	2.2	0.62
25. I’ve been thinking hard about what steps to take.		4 (1.8)	36 (16.4)	87 (39.5)	93 (42.3)		
18. I’ve been making jokes about it.	Humour	103 (46.8)	76 (34.5)	33 (15.0)	8 (3.6)	0.56	0.59
28. I’ve been making fun of the situation.		157 (71.4)	48 (21.8)	12 (5.5)	3 (1.4)		
20. I’ve been accepting the reality of the fact that it has happened.	Acceptance	28 (12.7)	97 (44.1)	67 (30.5)	28 (12.7)	1.48	0.68
24. I’ve been learning to live with it.		20 (9.1)	93 (42.3)	80 (36.4)	27 (12.3)		
22. I’ve been trying to find comfort in my religion or spiritual beliefs.	Religion	37 (16.8)	117 (53.2)	52 (23.6)	14 (6.4)	1.07	0.65
27. I’ve been praying or meditating.		56 (25.5)	127 (57.7)	30 (13.6)	7 (3.2)		
13. I’ve been criticizing myself.	Self-blame	46 (20.9)	115 (52.3)	55 (25.0)	4 (1.8)	1.07	0.61
26. I’ve been blaming myself for things that happened.		48 (21.8)	116 (52.7)	51 (23.2)	5 (2.3)		

M—mean, SD—standard deviation.

**Table 5:** Detailed characteristics of the results of the EEP-10.

Number of Item	Never		Almost Never		Sometimes		Quite Often		Very Often	
How often in thelast month:	N	%	N	%	N	%	N	%	N	%
were you upset, because something unexpected happened?	1	0.45%	16	7.27%	93	42.27%	83	37.73%	27	12.27%
did you feel that important matters inyour life had slipped out of control?	5	2.27%	40	18.18%	120	54.55%	50	22.73%	5	2.27%
did you feet nervousness andtension?	0	0.00%	8	3.64%	105	47.73%	68	30.91%	39	17.73%
were youconvinced that you were able to deal with personal problems?	1	0.45%	12	5.45%	78	35.45%	70	31.82%	59	26.82%
did you feel things were piling up in your mind?	0	0.00%	12	5.45%	91	41.36%	69	31.36%	48	21.82%
did you state that you were not dealing with everyone’s duties?	4	1.82%	51	23.18%	128	58.18%	28	12.73%	9	4.09%
could you master your irritability?	0	0.00%	14	6.36%	58	26.36%	40	18.18%	108	49.09%
did you feel that you were leaving everything?	2	0.91%	15	6.82%	100	45.45%	92	41.82%	11	5.00%
did you get angry because you had no impact on what happened?	1	0.45%	25	11.36%	136	61.82%	45	20.45%	13	5.91%
did you feelyou could not overcomeincreasing difficulties?	2	0.91%	30	13.64%	135	61.36%	42	19.09%	11	5.00%

**Table 6:** Impact of gender, place of residence and place of work on stress coping strategies.

	Gender		Place of Residence		Place of Work	
	U	*p*	U	*p*	U	*p*
Self-distraction	3423	0.493	5150.5	0.89	5957.5	0.855
Active coping	3018.5	0.068	4984.5	0.596	5179.5	0.059
Denial	3459	0.556	5165	0.917	5269.5	0.092
Substance use	2848	0.003*	4953	0.431	5272	0.028 *
Use of emotional support	3294.5	0.292	4635.5	0.175	4827	0.008 *
Use of instrumental support	3281.5	0.278	5045	0.700	5558.5	0.292
Behavioral disengagement	3415	0.478	5110	0.816	5191	0.064
Venting	3572	0.783	4605.5	0.153	5692	0.442
Positive reframing	3451	0.539	4991	0.606	5576	0.308
Planning	3094	0.107	5164	0.915	5093.5	0.038 *
Humour	3517.5	0.665	4355	0.041	5912.5	0.774
Acceptance	3629.5	0.911	4907	0.478	5796	0.592
Religion	3614.5	0.876	5028.5	0.665	5958.5	0.853
Self-blame	3490.5	0.614	5023	0.659	5695	0.446

U—value of the Mann–Whitney U statistics, *p*—significance level, *—significance level is less than 0.05.

**Table 7:** Impact of age, education, and work experience on stress coping strategies.

	Age		Education		Work Experience	
	H	*p*	H	*p*	H	*p*
Self-distraction	0.99	0.803	5.06	0.08	5.85	0.21
Active coping	2.68	0.443	1.64	0.44	3.43	0.488
Denial	2.69	0.442	2.28	0.321	4.45	0.349
Substance use	5.08	0.166	2.59	0.274	6.26	0.18
Use of emotional support	0.89	0.827	1.25	0.537	2.44	0.656
Use of instrumental support	1.25	0.741	0.57	0.752	1.45	0.836
Behavioral disengagement	1.94	0.585	0.93	0.628	4.95	0.292
Venting	5.71	0.127	1.83	0.401	8.12	0.087
Positive reframing	4.3	0.231	0.83	0.66	5.97	0.202
Planning	2.14	0.543	1.89	0.389	4.91	0.297
Humour	0.62	0.892	5.65	0.059	0.87	0.929
Acceptance	0.51	0.916	2.14	0.343	4.02	0.404
Religion	4.64	0.201	4.25	0.119	11.47	0.022 *
Self-blame	0.36	0.947	2.34	0.31	1.51	0.825

H—Kruskal–Wallis test, *p*—significance level, *—significance level is less than 0.05.

## Data Availability

Data are available upon reasonable request.

## Public Involvement Statement

No public involvement in any aspect of this research.

## Guidelines and Standards Statement

This manuscript was drafted against the (STROBE) for a cross-sectional study.
